# Supplementary material for: Content-rich biological network constructed by mining PubMed abstracts
Source: BMC Bioinformatics. 2004 Oct 8;5:147. doi: 10.1186/1471-2105-5-147 (PMC528731; doi:10.1186/1471-2105-5-147)
Supplement: Additional File 5 — The original Chilibot query results of the term "long-term potentiation (LTP)" and 22 other terms, limiting the latest references analyzed to the years 1990, 1995, 2000, and 2004. [file 1471-2105-5-147-S5.bz2 › chilibotAdditionalFile5/ltp2000/html/SYNAPSIN I_ERK.html]

 


 **SYNAPSIN I** and **ERK** 
  
Found 1 abstracts in PubMed,  **1 abstracts were retrieved and analyzed**.  


---

 Search Google  |
 PDF files only 
|  EDU domain only 

---

**Interactive relationship** (e.g. stimulation, inhibition, etc)

**Parallel relationship** (e.g. studied together, co-existance, homology, etc.)

- these effects are accompanied by a stimulation of STAT3 tyrosine phosphorylation, an inhibition of MAPK  **ERK**  activity, a decreased phosphorylation of the presynaptic MAPK  **ERK**  substrate  **synapsin I**  and no detectable effects on SAPK JNK.  Ref: 10762353 Eur J Neurosci, 2000
